# A taxonomic review of Eucalantica Busck (Lepidoptera, Yponomeutidae) with descriptions of six new species

**DOI:** 10.3897/zookeys.118.956

**Published:** 2011-07-13

**Authors:** Jae-Cheon Sohn, Kenji Nishida

**Affiliations:** 1 Department of Entomology, University of Maryland, College Park, MD 20742, USA; 2 Escuela de Biología, Universidad de Costa Rica, 2060 San José, Costa Rica

**Keywords:** Costa Rica, *Eucalantica*, lectotype, Mexico, new species, taxonomy, USA, *Vaccinium*, Yponomeutoidea

## Abstract

The New World genus *Eucalantica* Busck, 1904 is reviewed. It comprises seven species, six of which are described as new: *Eucalantica costaricae* Sohn & Nishida, **sp. n.**, *Eucalantica ehecatlella* Sohn & Nishida, **sp. n.**, *Eucalantica icarusella* Sohn & Nishida, **sp. n.**, *Eucalantica powelli* Sohn, **sp. n.**, and *Eucalantica pumila* Sohn, **sp. n.**, all five from Costa Rica; *Eucalantica vaquero* Sohn, **sp. n.** from southern USA and Mexico. The type species, *Eucalantica polita* (Walsingham, 1881), is redescribed and a lectotype and two paralectotypes are designated. Illustrations and keys based on the forewing patterns and the genitalia of each sex are provided. Our review suggests that there remains an undiscovered high diversity of *Eucalantica* in the tropical highlands of Central America.

## Introduction

The genus *Eucalantica* was proposed by Busck (1904) to account for differences of the type species *Calantica polita* Walshingham, 1881, from other *Calantica* Zeller, 1847, a junior homonym of *Calantica* Gray, 1825, whose replacement name is *Niphonympha* Meyrick, 1914. Busck suggested that *Eucalantica* is distinguished from *Niphonympha* in having scale tufts on the 3rd segment of labial palpus, Rs1 (=R2) and Rs2 (=R3) stalked in the forewings, and CuA2 arising near the middle of the cell in the hindwings. These characteristics are, however, homoplasious over the yponomeutoid lineages. Only their combination may help in distinguishing *Eucalantica* from other genera. The taxonomic position of the genus remains uncertain. Kyrki (1990) placed *Eucalantica* in Yponomeutidae without explanation. We follow this tentative placement. To establish which apomorphic characters define *Eucalantica*, a phylogenetic analysis of the Yponomeutoidea would be necessary but is beyond the scope of this work.

*Eucalantica* has been regarded as monobasic since the original description. Powell and Opler (2009) first challenged this status when mentioning the presence of species similar to *Eucalantica polita* from the high-elevation forests of Mexico and Costa Rica but they did not describe them. This discovery raised the possibility that the genus is more diverse and widely distributed than previously thought.

The purpose of this paper is to describe six new species of *Eucalantica*, five of which were from Costa Rica and one from southern USA and Mexico, to revise the definition of the genus and to present identification keys to adults based on external features and genitalia.

## Materials and methods

Pinned specimens from six institutional collections were examined. The abbreviations of these depositories are as follows:

BMNHNatural History Museum (formerly British Museum of Natural History), London, UK;
            

EMECEssig Museum of Entomology, University of California, Berkeley, USA;
            

INBIOInstituto Nacional de Biodiversidad, Santo Domingo de Heredia, Costa Rica;
            

MCZMuseum of Comparative Zoology, Harvard University, Cambridge, USA;
            

UCRMuseo de Zoología, Escuela de Biología, Universidad de Costa Rica, San José, Costa Rica;
            

USNMNational Museum of Natural History (formerly United States Museum of Natural History), Washington DC, USA.
            

Specimen label data are arranged by country, following in order of sex, state/province, specific locality, collecting date, collector and rearing records, if pertinent, and ending with specimen depository in parenthesis. The individuals whose sex cannot be determined are listed as ‘ex'.

Selected specimens were dissected for genitalia and abdominal structures, following Clarke (1941), except that chlorazol black was used for staining. Dissected genitalia were mounted on the microscope slides in Euparal resin (BioQuip Products Inc.). The genitalia slide numbers (GSN) are given for the dissected specimens with the suffix ‘USNM' for USNM specimens, ‘EMEC-JCS' for EMEC specimens and ‘SJC' for INBIO specimens. Unmounted genitalia are stored in glycerin-filled, transparent envelopes which are attached with dissected specimens. Pinned specimens were examined under a Leica MZ APO stereoscope. Slide-mounted specimens were examined under a Leica LETTZ-DMRX microscope.

Terms for genitalia and wing venation follow Klots (1970) and Wootton (1979), respectively. The 7th, 8th, and 9th abdominal segments are abbreviated as A7, A8, A9 respectively in the descriptions; the 7th and 8th sternite of females as S7 and S8.

## Taxonomic accounts

### Genus Eucalantica

Busck, 1904

http://species-id.net/wiki/Eucalantica

**Figures 1–4.**
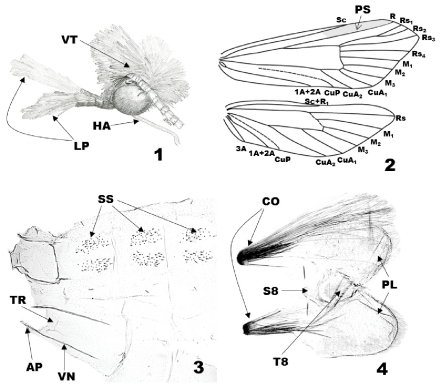
Generic characteristics of *Eucalantica*. **1** head of *Eucalantica polita* (lateral view) **2** wing venation of *Eucalantica polita* (gray shade – pterostigma) **3** abdominal segment II-IV of *Eucalantica costaricae* sp. n. **4** abdominal segment VIII of *Eucalantica costaricae* sp. n. Abbreviations: AP = apodeme; CO = coremata; LP = labial palpus; HA = haustellum; PL = pleural lobe; PS = pterostigma; S8 = eighth sternite; SS = spiniform setae; T8 = eighth tergite; TR = transverse ridge; VN = venula.

Figs 1 [Fig F2] [Fig F3] [Fig F4] [Fig F5] [Fig F6] –35

Eucalantica Busck, 1904: 750. Type species: *Calantica polita* Walsingham, 1881, by original designation and monotypy.

#### Diagnosis.

This genus is superficially similar to *Thecobathra* Meyrick, 1922, which also has a silvery white body and forewings, but differs from the latter in having a dark brown costal streak in forewing. The male genitalia of *Eucalantica* are distinguished from those of *Thecobathra* in having three or four spines on socii (none or one spine in the latter) and a lack of dentiform projections on phallus (present in the latter). The female S8 is entirely or almost entirely sclerotized in *Eucalantica*, but not in *Thecobathra*. The female genitalia of those two genera are also different in the shape of the signum, if present: keel-like or discoid plate in *Eucalantica*, cruciform in *Thecobathra*.

#### Description.

**Figures 5–14. F2:**
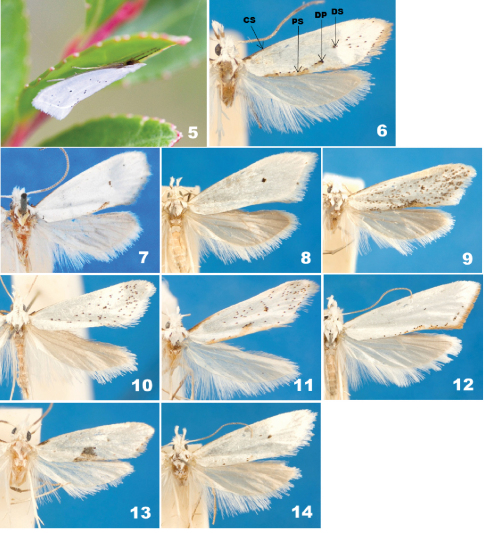
Adults of *Eucalantica*. **5** *Eucalantica costaricae* sp. n., resting on underside of *Vaccinium floribundum*, Cerro de la Muerte, Costa Rica **6** *Eucalantica ehecatlella* sp. n. (male, holotype) **7–9** *Eucalantica polita* **7** lectotype (male) **8** pale variant (female) **9** maculate variant (male) **10** *Eucalantica costaricae* sp. n. (female, paratype) **11** *Eucalantica icarusella* sp. n. (male, holotype) **12** *Eucalantica powelli* sp. n. (female, paratype) **13** *Eucalantica pumila* sp. n. (male, holotype) **14** *Eucalantica vaquero* sp. n. (female, paratype). Abbreviations: CS = costal streak; DP = dorsal patch; DS = discal spot; PS = posterior suffusion.

When resting, *Eucalantica* moths lay their body parallel to the substrate with their forelegs extended forward (Fig. 5).

#### Head.

(Fig. 1). Vertex vestiture rough with white, piliform scales; frons dark brown. Antennae filiform, 3/5 as long as forewing; scape white, with brown pecten; pedicel and first two flagellomeres with two complete whorls of scales per segment, white dorsally, gray ventrally; the remaining flagellomeres with a dorsal cover of gray scales on anterior half, a complete whorl of gray scales on distal half. Labial palpus porrect, 1st segment pale brownish gray, 1/4 as long as 2nd; 2nd segment dark brown, with denser scales distad, as long as eye diameter; 3rd segment white except dark brown on ventrobasal area, with white scale tufts dorsally, as long as 2nd. Maxillary palpus 4 segmented. Proboscis devoid of scales, longer than labial palpus.

#### Thorax and abdomen.

Tegula and mesonotum white. Foreleg lustrous dark brown dorsally, gray ventrally; epiphysis arising at middle. Midleg with coxa to tibia lustrous pale brown dorsally, silvery white ventrally; first tarsus dark brown dorsally, silvery white ventrally; the remaining tarsi brownish gray with dark brown ring on distal end. Hindleg silvery white, slightly tinged with pale brown ventrally. The forewings (Figs 6–14) white, elongate-triangular, costa straight, apex at anterior 1/3 of termen, obtuse-angled, termen oblique after apex; a black spot at the upper corner of discal cell; scattered black spots on the posterior 1/2 and distal 2/3; a brown or orange dorsal patch; however, the latter two are often reduced, depending on the individual. The forewing venation of *Eucalantica* (Fig. 2) with pterostigma 2/5 of costa before R; Rs1 and Rs2 stalked; Rs4 below apex; M2 and M3 at base closer than M1; CuA1 directed to tornus; CuA2 ending at posterior margin. The hindwing slightly broader than forewing, pale gray, darkened to apex and anterior margin, termen broadly round, apex narrowly round; venation (Fig. 2) with Sc+R1 ending at the middle of anterior margin; Rs directed to apex; M1, M2 and M3 evenly spaced; CuP close to 1A+2A. Abdomen silvery white, slightly tinged with pale brown on basal half; pleural lobe silvery white.

#### Abdominal sternum II and terga.

(Fig. 3). Apodeme slender, 1/5 as long as venula; venula slender, 4/5 as long as 2nd sternite; transverse ridge at posterior 1/6 of 2nd sternite. A pair of spiniform setal zones on tergum II~VII; in *polita*, paired zones expanded, fused with each other.

#### Male A8.

(Fig. 4). A pair of coremata twice as long as pleural lobes; tergite elliptical, with lanceolate caudal end; pleuron expanded posteriorly as lobate; sternite subtriangular, enlarged caudad, posterior margin concave.

#### Male genitalia.

(Figs 17–30). Uncus linguiform, convex posteriorly, medially fused with tegumen; in four of the seven species, a pair of lateral humps present near apex; socii elongate, extended from ventrobasal area of uncus, with a row of 3–4 spines ventroterminally. Tuba analis with weakly sclerotized area ventrally (‘subscaphium'), continuous to gnathos; gnathos as a transverse bulge below tuba analis, with narrow, band-like sclerotization along apical edge. Valva obovate or rectangular, setose on the posterior half of the ventral side, with species-specific groove or projections above basal sacculus. Vinculum narrower to saccus; saccus elongate. Aedeagus straight or bent medially; cornuti absent or as a zone of minute spinules.

#### Female genitalia.

(Figs 31–35). Papillae anales subtriangular. A pair of hairy humps on the distal margin of S8; interspace between the humps with dense, minute thorns, the thorny area extending above and below S8 humps. Segment S8 entirely or mostly sclerotized, sometimes posterolateral margins forming a semicircular fold (Figs 32 and 35, indicated by asterisk); depending on the species, with a pair of pits (Fig. 35a) or semicircular depression (Fig. 31b) near ostium. Antrum digitate or bowl-shaped, with numerous minute thorns internally; thorny area extending caudally beyond ostium bursae. Ductus seminalis near a connection between ductus and corpus bursae; bulla seminalis as large as (in *polita*) or smaller than corpus or absent (in *costaricae*). Corpus bursae very fragile due to its thin wall; signum absent in two species, present in three species and shaped like a dentate keel or a small scobinate disk.

#### Species diversity.

The distribution of *Eucalantica* as shown in this paper indicates a high diversity of the genus in the Central America. Three of the five Costa Rican species described in this paper were found in the high-elevation oak forests of Cerro de la Muerte region, indicating that multiple species can coexist in a single ecozone. Interestingly, there exists a different group of congeners in the high mountains of Heredia province. This pattern predicts more undescribed species of *Eucalantica* present along the montane systems of Costa Rica and other Central American countries.

#### Key to the adults of *Eucalantica* species including variants in forewing patterns

Note: External appearance is usually inadequate for species identification of *Eucalantica*. Whenever possible, examination of the genitalia is advised for reliable identifications of the species.

**Table:** 

1	No patch and suffusion on dorsal area of forewing	2
–	Dorsal patch and/or suffusion on forewing present (Fig. 6)	3
2	Black spots scattered on forewing	*Eucalantica costaricae* sp. n.
–	Forewing almost immaculate (except discal spot)	*Eucalantica polita*(Fig. 8)
3	Dorsal patch on forewing without posterior suffusion	4
–	Dorsal patch on forewing with posterior suffusion (Fig. 6)	6
4	Dorsal patch bar-like	5
–	Dorsal patch triangular	*Eucalantica pumila* sp. n.
5	Terminal half of fringe pale grayish brown on forewing	*Eucalantica polita* (Fig. 7)
–	Terminal 1/4 of fringe pale grayish brown on forewing	*Eucalantica vaquero* sp. n.
6	Posterior suffusion extending along entire dorsal margin of forewing	7
–	Posterior suffusion only on basal 2/3 of dorsal margin of forewing	*Eucalantica ehecatlella* sp. n.
7	Posterior suffusion with apparent dorsal patch	8
–	Posterior suffusion with reduced dorsal patch	*Eucalantica powelli* sp. n.
8	Black spots on forewing sparse and covering only distal third	*Eucalantica icarusella* sp. n.
–	Black spots on forewing dense and scattered over entire surface	*Eucalantica polita*(Fig. 9)

#### Key to *Eucalantica* species based on male genitalia

**Table:** 

1	A pair of lateral lobes near uncus apex present	2
–	A pair of lateral lobes near uncus apex absent	5
2	Aedeagus with swelling at 3/5 (Figs 24e and 28e)	3
–	Aedeagus without swelling	4
3	Apex of uncus medially markedly convex (Fig. 27a)	*Eucalantica vaquero* sp. n.
–	Apex of uncus medially nearly flat (Fig. 23a)	*Eucalantica icarusella* sp. n.
4	Valva with a triangular mound above subbasal saccular region (Fig. 17c)	*Eucalantica polita*
–	Valva without a mound above subbasal sacculus region (Fig. 25c)	*Eucalantica powelli* sp. n.
5	Valva obovate	6
–	Valva with costal and saccular margin parallel each other in most areas	*Eucalantica pumila* sp. n.
6	Base of valva with two arched grooves (Fig. 21c)	*Eucalantica ehecatlella* sp. n.
–	Base of valva with one arched groove (Fig. 19c)	*Eucalantica costaricae* sp. n.

#### Key to Eucalantica species based on female genitalia

Note: the females of *Eucalantica ehecatlella* and *Eucalantica pumila* are unknown

**Table:** 

1	Signum present	2
–	Signum absent	4
2	Signum keel-shaped (Figs 34c, 35c)	3
–	Signum discoid (Fig. 33c)	*Eucalantica costaricae* sp. n.
3	A pair of pits present on S8 around ostium bursae (Fig. 35a)	*Eucalantica icarusella* sp. n.
–	S8 without pits	*Eucalantica vaquero* sp. n.
4	S8 with a pair of semicircular folds posterolaterally (Fig. 32)	*Eucalantica powelli* sp. n.
–	S8 without semicircular fold	*Eucalantica polita*

### Eucalantica polita

(Walsingham, 1881)

http://species-id.net/wiki/Eucalantica_polita

Figs 1–2 7–9 17–18 31

Calantica polita Walsingham, 1881: 302, pl. 35: 2.
Eucalantica polita ; Busck, 1904: 750.

#### Types examined.

**Lectotype** ♂ (here designated; Fig. 7) – USA: “Lectotype [on a round paper with cobalt blue border]", “Lake Co./ CALIFORNIA/ 17–19 1871/ Wlsm. [on a rectangular paper]", “Walsingham/ Collection/ 1910–427 [on a rectangular paper]", “Calantica/ polita Wlsm/ P.Z.S.Lond.p.302.tf.35'2 1881/ TYPE ♂ [on a rectangular paper with black margins]", BMNH. **Paralectotypes** 2♂ – USA: “Calantica polita Wl. Cala. [California], Pr.Z.S.1881.p302/ pl.35.f.2 [handwriting on a rectangular paper]", “Type 14992 [in a red rectangular paper]", “Wlsm. To Chamb. [handwriting on single line paper]", MCZ. Walsingham (1881) did not state the exact type locality and the number of specimens for his description of *Calantica polita*. A male specimen from BMNH has a red-bordered round label written “Type". Two type specimens of *Calantica polita* from MCZ are duplicates by Walsingham which were sent to Chambers (Miller and Hodges 1990). Therefore, all three specimens from BMNH and MCZ which hold “Type" label must be syntypes as Miller and Hodges (1990) already indicated. We formally designate a lectotype of *Calantica polita* amongst these specimens.

#### Specimens examined.

CANADA: 2♂, British Columbia, Vancouver, BC, 3 August 1902 (USNM); 1♂, British Columbia, Vancouver Isl., Wellington, 14 April 1902 (USNM); 3♂, ditto, February 1905, GW Taylor (USNM); 1♂, ditto, 27 April 1904, T Bryant (USNM); 1M, ditto, October 1905, GW Taylor (USNM); 3♂, ditto, November 1905, GW Taylor (USNM); 3♂, ditto, no date, GW Taylor (USNM); 1♂, British Columbia, Vancouver Is., Duncans, 12 April 1892, Hanham (USNM); 1♂, ditto, June 1908, Hanham (USNM); 1♂, ditto, 5 October 1908, Hanham (USNM); 1♂, ditto, April 1909, Hanham (USNM); 1ex, ditto, no date, Hanham (USNM); 1♂1♀, British Columbia, Departure Bay, Bio Station, April 1909 (USNM); 1♂, British Columbia, Goldstream, 18 April 1921, EH Blackmore (USNM). USA: 1♂, Washington, Goldbar, 25 September 1983, DF Bray (USNM); 1♂, Washington, Long Beach, Clarke's Nursery, 24 July 1965, EP Breakey, “reared from *Vaccinium ovatum*", GSN [USNM-77947] (USNM); 1♂, Washington, Long Beach, 10 December 1964, EP Breakey & EG Tinius, “from larva boring rhododendron twig, emerged on 31 December 1964" (USNM); 2♂2♀, Washington, Tacoma, 20 May 1928, M Clarke (USNM); 1♂, Washington, Lake Crescent, June 1971, EC Zimmerman (USNM); 1ex, Washington, Tiago, 17 June 1918, HK Plank, “on huckberry [sic]/ winter" (USNM); 1♂, Washington, Seattle, 27 May 1901, GSN [USNM-91608] (USNM); 1♂, ditto, 12 October 1923, JFG Clarke (USNM); 1♂, ditto, 20 April 1931, WMW Baker, “leaf miner in rhododendron" (USNM); 1♂, Washington, Olympic Mts., 12 April 1892 (USNM); 6♂, Washington, Olympic Mts., Barnes Creek, 5–6 August 1936, AF Braun (USNM); 1♂, Washington, Olympic Mts., Hurricane Ridge, alt. 3000ft, 15 June 1955, JFG Clarke (USNM); 2♂, Washington, Harstine Island, 24 July 1960, EP Breakey, “from larvae (leaftiers) feeding in tips of huckleberry" (USNM); 1♀, Washington, Hoquiam, Burke Colr., 6 May 1904, Fivino (USNM); 3♂1♀, Washington, San Juan Co., Deer Harbor, Orcas Island, 14 July 2002, J Powell (EMEC); 1♂, Washington, Kitsap Co., Bainbridge Island, Venice District, 3–4 April 2001, J Powell (EMEC). 3♂1♀, Oregon, NW corner Douglas Co., Lake Tahkenitch, 26 August 1969, J Powell (EMEC); 1♂, ditto, [no date & collector info] (EMEC); 1♀, Oregon, Coos Co., Bullards Beach, 2 mi N from Bandon, 24–25 August 1969, J Powell (EMEC).1♂♀, California, San Francisco, Big Basin, 18 June 1971, E Jäckh (USNM); 1♂, California, Del Norte Co., Redwoods, 23 August 1936, AF Braun (USNM); 1♂, California, Humboldt Co., Fieldbrook, 18 May 1903, HS Barber (USNM); 1♂, ditto, 26 May 1903, HS Barber (USNM); 1♂, California, Humboldt Co., 4 mi S from Fieldbrook, 29 June 1969, J Powell (EMEC); 11♂16♀, California, Humboldt Co., 11 mi NE from Blue Lake, Redwood Summit, 9 May 1961, J Powell (EMEC); 1♂, California, Monterey Co., Big Creek Reserve, 8–9 June 2001, J Powell (EMEC); 1♀, ditto, 21–22 July 1992, B Scaccia & R Zuniga (EMEC); 12♂28♀, California, Monterey Co., Big Creek Reserve, Devils Cr. Flat, alt. 120m, Redwood riparian, 23–25 April 1987, J Powell, “JAP no. 87D29: emerged in 16–21 May 1987, reared from *Vaccinium ovatum*" (EMEC); 6♂, California, Humboldt Co., Kneeland, 69 Prairie Lane, 12–14 March 2001, RS Wielgus, GSN [USNM96387] (USNM); 14♂11F, ditto, 18–20 March 2001, RS Wielgus (EMEC); 1♂, California, Humboldt Co., Arcata, 24 June 1969, J Powell (EMEC); 1♂, ditto, 28 June 1969, J Powell (EMEC); 1♀, California, Humboldt Co., Richardson Grove St. Park, 18 June 1962, CA Toschi (EMEC); 1♂, California, Marin Co., Inverness Ridge, 15 May 1970, J Powell (USNM); 3♂, ditto, alt. 100m, 21–24 May 1995, JA Powell (EMEC); 1♂2♀, ditto, alt. 40–250m, 20 October 1999, JA Powell (EMEC); 1♀, ditto, alt. 270m, 19 September 1998, JA Powell (EMEC); 1♂, ditto, alt. 250–300m, 19–20 May 1998, JA Powell (EMEC); 1♂, California, Marin Co., Inverness Park, alt. 150m, 26–30 September 1999, J Powell (EMEC); 1♂, ditto, 8–14 October 1999, J Powell (EMEC); 1♂1♀, ditto, 15–22 October 1999, J Powell (EMEC); 1♂, ditto, alt. 175m, 13–19 2003, J Powell (EMEC); 1♂, ditto, 20–26 October 2003, J Powell (EMEC); 2♂, California, Marin Co., 2mi SE Inverness Ridge, alt. 700–1100ft, 15–16 May 1970, RE Dietz (EMEC); 1♀, California, Marin Co., Mt. Vision, Inverness Ridge, 24 April 1982, JA Powell, “JAP no. 82D46: reared from *Vaccinium ovatum*" (EMEC);1♀, California, Marin Co., Palomarin, 7–8 May 1990, P Super (EMEC); 1♂2♀, California, Marin Co., Alpine Lake, alt. 250–350m, 11 April 1992, J Powell, “JAP no. 92D39.1: emerged on 8 May 1992, reared from *Vaccinium ovatum*" (EMEC); 3♂1♀, California, Tomales Bay, Marin Co., 21 January 1959, J Powell (EMEC); 1♂, ditto, 17 February 1961, J Powell (EMEC); 14♂10♀, California, San Mateo Co., San Bruno Mt., 13 April 1981, JA DeBenedictis, “JADeB no. 81103-A: emerged in 6–12 May 1981, reared from *Vaccinium ovatum*" (EMEC); 1♂, ditto, 16 May 1984, JB Whitfield & JA DeBenedictis, “JBW no. 84E31: emerged between 25 May & 4 June 1984, reared from *Vaccinium ovatum*" (EMEC); 1♂1♀, California, San Mateo Co., San Bruno Mt., Radio Tower Road, 16 May 1984, JA Powell, “JAP no. 84E31: emerged on 4 June 1984, reared from *Vaccinium ovatum*" (EMEC); 1♀, California, Sonoma Co., 10–25 May, AH Vachell (USNM); 1♂1♀, California, Sonoma Co., Salt Point St. Park, 20 July 1990, RJ Robertson (EMEC); 4♂5♀, California, Mendocino Co., 2 mi S from Rockport, 1 February 1962, J Powell (EMEC); 1♂1♀, California, Mendocino Co., 5 mi NW from Comptche, Pygmy Forest, 10 April 1981, JA DeBenedictis, “JADeB no. 8100-A: emerged on 29 April & 4 May 1981, reared from *Vaccinium ovatum*" (EMEC); 4♂, California, Del Norte Co., 8 mi N from Klamath, Damnation Cr., 20 July 1969, DP Levin (EMEC); 1♂, California, Santa Barbara Co., Santa Cruz Is., Ridge N of Laguna Canyon, 28 April 1966 (EMEC); 1♀, California, Santa Barbara Co., Santa Cruz Is., Canada de la Cuesta, 15 March 1969, J Powell, “JAP no. 69C39: emerged on 4 April 1969, reared from *Vaccinium ovatum*" (EMEC); 1♀, California, Santa Barbara Co., Santa Cruz Is., Felton, 20–21 July 1991, J Powell (EMEC); 1♂, [no specific locality], 1882, Walsingham, GSN [USNM-91607] (USNM); 1♂, ditto, [no date], Fernald (USNM).

#### Diagnosis.

This species externally resembles *Euceratia castella* Walsingham, 1881, among the described species of North America, but is easily distinguished from the latter in having a dorsal patch on forewings and by in lacking white annulations on the antennae.

#### Redescription.

(Figs 7–9). Forewing length 5.5–8mm (mean=7.19mm, n=58); basal 1/4 of costa dark brown; an oblique, bar-like, reddish brown patch on distal 1/3 of posterior margin, surrounded by black speckles; posterior suffusion reddish brown, as long as dorsal patch; posterior suffusion and/or dorsal patch lost and black specks peppering, depending on the individuals; a black spot at the end of discal cell; a black scale on each vein along termen; fringes white on basal half, grayish brown on distal half, or entirely white in some specimens. Hindwing anterior margin 2× longer than maximum width; fringe pale gray on basal half, white on distal half.

#### Male genitalia.

(Figs 17, 18) (6 preparations examined). Uncus (Fig. 17a) linguiform, convex posteriorly, with a pair of short, digitate tubercle posterolaterally; socii digitate, as long as saccus, with a row of 4 or 5 short ventral spines terminally, gradually smaller from basal to terminal spine (Fig. 17b). Tegumen parallel-sided; subscaphium (Fig. 17d) strongly bulged ventrad. Valva obovate, saccular margin evenly rounded, 2× longer than tegumen; costa curved at 1/4, narrowly sclerotized in basal 1/4; a small triangular mound above basal 2/5 of saccular margin (Fig. 17c). Saccus slender, as long as socius. Aedeagus (Fig. 18) slender, 3× length of saccus, weakly sinuate; cornutus absent.

#### Female genitalia.

(Fig. 31) (5 preparations examined). S8 sclerotized, with a shallow bulge posterior to S8 humps; minute thorns on the bulge; semicircular depression anteriolaterally (Fig. 31b). Apophysis posterioris 2× longer than papillae anales, 2.5× longer than apophysis anteroris excluding basal Y-fork; longer branch of the Y-fork 1.2× longer than shorter branch or apophysis anterioris. Minute thorns on area between S8 humps and ostium bursae. Ductus bursae as long as apophysis posterioris; antrum in posterior 1/5 of ductus bursae, digitate, broadened at ostium, with minute thorns on internal wall (Fig. 31a); bulla seminalis as large as corpus bursae. Corpus bursae ovoid; signum absent.

#### Distribution

**Figure 15. F3:**
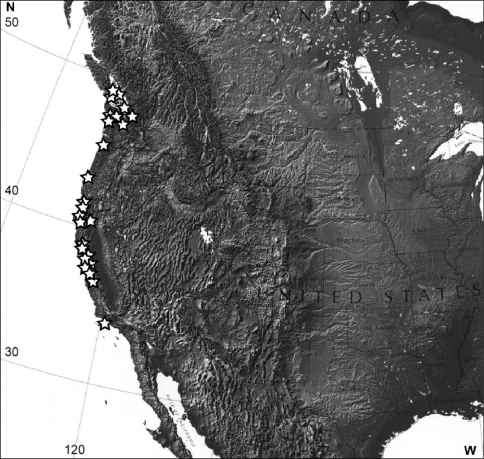
Distribution of *Eucalantica polita* (white star). Map from www.nationalatlas.gov.

(Fig. 15). Pacific side coastal regions of Canada (British Columbia) and United States (Washington, Oregon, California).

#### Host plant.

The larvae feed on flowers and leaves of California Huckleberry, *Vaccinium ovatum* Pursh(Ericaceae) (Powell & Opler, 2009). In the USNM collection, there exist two specimens of *Eucalantica polita* reared from “rhododendron", possibly *Rhododendron pacificum*. These records, however, need to be confirmed. The host record “huckberry" from USNM must be an error for “huckleberry". The label data available from museum specimens indicate that the larvae are twig-borers, leaf-miners or leaf-tiers. The larvae of *Eucalantica polita* are primarily external feeders which web amongst inflorescences or young vegetative terminals of *Vaccinium ovatum* (Jerry Powell, personal communication). All records of the internal feeding larvae of *Eucalantica polita* are associated with “rhododendron", a host which is yet unverified.

#### Remarks.

*Eucalantica polita* shows continuous variations in forewing patterns between two extremes which are very reduced (Fig. 8) or maculate throughout (Fig. 9). Those variants coexist temporally and spatially, for which no taxonomic consideration is necessary. However, some of the variants can be confused with the new species described in this study. Walsingham (1881) illustrated an individual of *Eucalantica polita* whose forewings have only a dorsal patch and discal spot (Fig. 7). We found that this variant is predominant (ca. 87%) amongst the specimens examined in our study. The maculate variants were the rarest (ca. 0.7%).

### Eucalantica costaricae

Sohn & Nishida sp. n.

urn:lsid:zoobank.org:act:9D5181DE-7F5C-47B1-AC4E-3F16C72A3CD5

http://species-id.net/wiki/Eucalantica_costaricae

Figs 3–4 10 19–20 33 

#### Type material.

**Holotype** ♂ – COSTA RICA: Cartago, El Guarco, Macizo de la Muerte, Sector de la esperanza, 9°46'14" N; 83°47'59"W, alt. 2600m, February 2002, R Delgado, BN-INB0003434063, GSN [SJC 640] (INBIO). **Paratypes** (5♂2♀) – COSTA RICA: 2♂, San José, Cerro de la Muerte, Villa Mills, La Georgina, 9°34'N; 83°43'W, alt. 3000m, 20 February 1999, K Nishida (USNM & UCR); 1♀, San José, Cerro de la Muerte, Estación Biológica de la UCR, 9°34'N; 83°45'W, alt. 3050m, 2 February 1999, K. Nishida (BMNH). 1♂, Cartago, Cerro de la Muerte, Georgina, 9°34'N; 83°45'W, alt. 3000m, 23–25 May 1985, J Powell & PA Opler (INBIO); 1♂, ditto (EMEC); 1♂, ditto, 20 June 1988, J Brown & J Powell (EMEC); 1♀, Cartago, Villa Mills, 9°34'N; 83°43'W, alt. 3000m, 3–4 July 1999, J Powell (EMEC).

#### Diagnosis.

This new species is superficially indistinguishable from some variants of *Eucalantica polita*. In such cases, examination of genitalia is necessary for reliable identification. *Eucalantica costaricae* differs from *Eucalantica polita* by the lack of lateral projections near the apex of the uncus in the male genitalia and in having a signum in the corpus bursae of the female genitalia.

#### Description

(Fig. 10). Forewing length 6.5–8mm (mean=7.48mm, n=9); posterior suffusion and dorsal patch absent; in majority of individuals, black spots scattered on distal and posterior half; fringes entirely white. In some specimens, all forewing pattern elements are lost except a discal spot. Hindwing anterior margin 3× longer than the maxium width; fringes entirely white.

#### Male genitalia.

(Figs 19, 20) (5 preparations examined). Uncus (Fig. 19a) linguiform apically; socii lunate, as long as saccus, long hairy dorsally, with four terminal spines in a row, third spine from tip longest, followed by second, fourth, and first in order of length (Fig. 19b). Tegumen subtriangular, 1.5× broader than uncus; subscaphium (Fig. 19d) appressed to tegumen. Valva obovate, 2.5× longer than socii, costa slightly incurved at basal 1/3; arched setose area above saccular base (Fig. 19c). Saccus very slender, as long as socius. Aedeagus (Fig. 20) attenuate in distal half, as long as and slightly wider than saccus, bent medially; carina slender, triangular; a zone of minute spinulate cornuti in distal half of aedeagus.

#### Female genitalia.

(Fig. 33) (2 preparations examined)**.** S8 sclerotized; minute thorns on semicircular area above S8 humps. Apophysis posterioris 2.5× longer than apophysis anterioris excluding basal Y-fork; longer branch of Y-fork 5× longer than shorter branch. Ductus bursae as long as corpus bursae; antrum in posterior 1/7 of ductus bursae, cup-shaped, with minute thorns on internal wall (Fig. 33a); bulla seminalis 2/3 as large as corpus bursae. Corpus bursae ellipsoid; signum as a small, scobinate disc (Fig. 33c).

#### Distribution.

Costa Rica (high elevations of Cerro de la Muerte of the Talamancan Mountain Range in Cartago and San José Provinces).

#### Habitat.

The adult specimens have been collected exclusively from the high elevation forests of Cerro de la Muerte where oaks are dominant below 3,300m (Zuchowski, 2007). See Nishida et al. (2002) for more details about the habitats. The second author (KN) observed one individual of this species resting on the underside of a leaf of *Vaccinium floribundum* Kunth (Fig. 1). Given the host association of *Eucalantica polita* with another *Vaccinium*, this plant is likely the larval host of *Eucalantica costaricae*.

#### Etymology.

The new species is named after Costa Rica, where the type locality is situated.

### Eucalantica ehecatlella

Sohn & Nishida sp. n.

urn:lsid:zoobank.org:act:0003EA08-8167-4DF1-BFC9-0B171C4835EE

http://species-id.net/wiki/Eucalantica_ehecatlella

Figs 6 21, 22 

#### Type material.

**Holotype** ♂ – COSTA RICA: Heredia, Volcán Barva, 6 km ENE from Vara Blanca, 10°10'34"N; 84°06'41"W, alt. 1950**–**2050 m, 16 February 2002, BN-INB0003220413, GSN [SJC 642] (INBIO). **Paratype** ♂ – COSTA RICA: same locality as holotype, 21 March 2002, A Kawahara, BN-INB0003217116, abdomen missing (INBIO).

#### Diagnosis.

This species is very close to *Eucalantica icarusella* in the shape of the dorsal patch of the forewing and in having entirely pale gray forewing fringes but differs from the latter by having most of the black dots sparsely scattered beyond the discal cell. *Eucalantica ehecatlella* is further distinguished from *Eucalantica icarusella* by the lack of projections near the apex of uncus in the male genitalia.

#### Description

(Fig. 6). Forewing length 5.0–6.2 mm (n=2) with dark brown costal streak in basal 1/4; posterior suffusion on basal half of dorsal margin, reddish brown with an intermittent, black line along upper border; black spots sparsely scattered on distal 1/4; terminal line narrow, black, intermittent; fringes pale orange, paler on tornus. Hindwing anterior margin 2× longer than maximum width; fringes pale gray.

#### Male genitalia.

(Figs 21, 22) (1 preparation examined)**.** Uncus (Fig. 21a) elongate, triangular, conical apically; socii bulged dorsally, sharp triangular in terminal 1/5, 1.5× longer than saccus, long-hairy dorsally, with four terminal spines in a row, third spine from tip longest, followed by second, fourth, and first in order of length (Fig. 21b). Tegumen parallel-sided; subscaphium (Fig. 21d) slightly bulged ventrad. Valva obovate, costal margin almost straight, apex broadly round; a semicircular emargination adjoining with a densely setose area and an oblique groove above saccular base (Fig. 21c); a subrectangular emargination near the middle of the base of valva ventrally (Fig. 21c). Saccus elongate, digitate, as long as uncus. Aedeagus (Fig. 22) of even width throughout, strongly curved medially, with a triangular carina terminally and a zone of minute-spinulate cornuti 1/3 as long as aedeagus.

#### Female.

unknown.

#### Distribution.

Costa Rica (Central Volcanic Range in Heredia Province).

#### Etymology.

The specific epithet is derived from ‘Ehecatl', a god of wind in Aztec mythology and refers to the windy habitat where the new species was collected.

### Eucalantica icarusella

Sohn & Nishida sp. n.

urn:lsid:zoobank.org:act:87237D85-EDA7-4668-ACF0-0D84ED2EB659

http://species-id.net/wiki/Eucalantica_icarusella

Figs 11 23–24 35 

#### Type material.

**Holotype** ♂– COSTA RICA: San José, Cerro de la Muerte, Estación Biológica de la UCR, 9° 34' N; 83° 45' W, alt. 3050m, 20 February 1999 (K. Nishida), GSN [USNM96397], USNM. **Paratypes** (6♂4♀) – COSTA RICA: 1♂, San José, Cerro de la Muerte, Estación Biológica de la UCR, 9°34'N; 83°45'W, alt. 3100m, 20 December 1998, K Nishida (BMNH); 2♂, San José, Cerro de la Muerte, Estación Los Nimbolos, 9°99'42.30"N; 83°44'27.2"W, alt. 3150m, 24–27 July 2006, JB Sullivan (USNM). 2♀, Cartago, Cerro de Muerte, Pension La Georgina, 9°34'N; 83°45'W, alt. 3000m, 23**–**25 May 1985, J Powell, GSN [EMEC-JCS 003] (EMEC); 1♀, 7km SE El Canon, 9°40'N; 83°55'W, 28 May 1985 (J Powell), GSN [EMEC-JCS 001] (EMEC). 1♂1♀, Alajuela, Volcán Poás, 10°11'00"N; 84°12'30"W, alt. 2550m, 6–7 June 1988 (J Brown & JA Powell), GSN [EMEC-JCS 004 (♀)] (EMEC). 1♂, Heredia, Volcán Barva, 6 km ENE from Vara Blanca, 10°10'34"N; 84°06'41"W, alt. 1950**–**2050 m, 20 March 2002, K Nishida, abdomen missing (USNM); 1♂, ditto, 12 April 2002, K Nishida (UCR).

#### Diagnosis.

This species is superficially similar to *Eucalantica costaricae*, but differs from the latter in having a posterior suffusion on the forewings and narrower hindwings. In the genitalia, *Eucalantica icarusella* is distinguished from *Eucalantica costaricae* in having projections (Fig. 23a) near the apex of the uncus in the males and having a pair of pits (Fig. 35a) near ostium bursae in the females.

#### Description

(Fig. 11). Forewing length 5.3–7.9 mm (mean=7.07mm, n=9); costal streak dark brown, broadly spread basally; dorsal patch at the middle of posterior margin, dentiform, orange, with a black line on upper border; posterior suffusion on basal 1/2 of dorsal margin, orange, with an intermittent black line on upper border; black spots peppering in distal 3/4, denser to distal 1/3; fringes pale gray in basal 1/3, brownish gray in distal 2/3. Hindwing anterior margin 2.5× longer than maximum width; fringes pale gray.

#### Male genitalia.

(Figs 23, 24) (3 preparations examined). Uncus (Fig. 23a) linguiform, apex slightly protruded, lateral lobes digitate, with transverse edge apically; socii digitate, narrowly round apically, as long as saccus, long-hairy dorsally, with three terminal spines in a row, all almost same in length (Fig. 23b). Tegumen as long as uncus, subtriangular posteriorly, parallel laterally in anterior half, enlarged in posterior half; subscaphium (Fig. 23d) appressed to tegumen. Valva elongate, almost of even width throughout, rounded apically, 3.5× longer than saccus; costa slightly bulged at basal 1/5; a semicircular emargination above saccular base, adjoining with a small tubercle at upper end (Fig. 23c). Saccus digitate, robust, broadened to base, as long as socius. Aedeagus (Fig. 24) almost straight, slightly bulged medially (Fig. 24e), 2.5× longer than saccus; a zone of minute-spinulate cornuti 2/5 as long as aedeagus.

#### Female genitalia.

(Fig. 35) (4 preparation examined). S8 sclerotized, quadrate, with a pair of semicircular, setose humps posteriorly; minute thorns on and posteriorn to S8 humps; semicircular, lateral pleats at the middle of S8 area (indicated with an asterisk in Fig. 35); a pair of pits adjacent to ostium (Fig. 35a). Apophysis posterioris 3.5× longer than apophysis anterioris excluding basal Y-fork; ventral branch of Y-fork fused with posterior margin of S8, dorsal branch 2× longer than apophysis anterioris, slightly sinuous. A zone of minute thorns extended from antrum to S8 pleats. Ductus bursae as long as corpus; antrum cylindrical, 1/6 as long as and 2× wider than ductus bursae, with minute thorns on internal wall (Fig. 35a); bulla seminalis 1/2 as long as ductus bursae. Corpus bursae oval, membranous, cervical area slightly protruding; signum keel-like with denticules on interior surface (Fig. 35c).

#### Distribution.

Costa Rica (high elevations of Cartago, Heredia and San José).

#### Etymology.

The new species is named after the Greek mythological character *Ikaros* (*Icarus* in Latin) and refers to the white forewing with scarlet dorsal suffusion resembling Icarus' waxy wings burnt down by sunlight.

### Eucalantica powelli

Sohn sp. n.

urn:lsid:zoobank.org:act:D7AF7609-BE3C-41CE-8F94-8039229CA82E

http://species-id.net/wiki/Eucalantica_powelli

Figs 12 25–26 32 

#### Type material.

**Holotype** ♂ – COSTA RICA: Cartago, Cerro de la Muerte, La Georgina, 9°34'N; 83°45'W, alt. 3000 m, 23–25 June 1985, J Powell & PA Opler, GSN [EMEC-JCS 012] (EMEC). **Paratypes** **(**1♂4♀**)** – COSTA RICA: 1♀, San José, 4.6 km E from Villa Mills, Sendero al Mirador, Est. Cuericí, 9°34'N; 83°43'W, alt. 2640m, 17–22 March 1996, A Picado, GSN [SJC 806] (INBIO). 1♂, Cartago,7 km SE El Cañón, 9°40'N; 83°55'W, alt. 2500 m, 28 May 1985, J Powell & JT Doyen (EMEC); 3♀, Cartago,Villa Mills, 9°34'N; 83°43'W, alt. 3000 m, 3–4 July 1999, J Powell, GSN [EMEC-JCS 002] (EMEC).

#### Diagnosis.

This new species is similar to immaculate variants of *Eucalantica polita* (Fig. 8) but differs from the latter in having posterior suffusion on entire dorsal margin of forewings. They are also distinguished by the male genitalia, i.e. triangular projection on valva closer to sacculus in *Eucalantica powelli*, and also by the female genitalia, i.e. the presence of posterolateral semicircular pleats (indicated with an asterisk in Fig. 32) in *Eucalantica powelli*.

#### Description

(Fig. 12). Forewing length 7.0–10.0 mm (mean=8.48mm, n=5); dorsal margin with a row of black dots from the base to the basal 1/3; posterior suffusion on distal 2/3 of dorsal margin, sinuate, orange, with an intermittent black line on upper boarder; terminal line on posterior half of termen, black, intermittent; fringes white in basal half, reddish brown in distal half.

#### Male genitalia.

(Figs 25, 26) (2 preparations examined). Uncus (Fig. 25a) elongate, subrectangular, as long as tegumen, with a pair of digitate lobes posterolaterally; socii digitate, as long as saccus, long-hairy dorsally, with four terminal spines in a row, third and fourth spines from top longest, followed by second, first in order of length (Fig. 25b). Tegumen parallel-sided; subscaphium (Fig. 25d) appressed to tegumen. Valva obovate; costa slightly curved at distal 1/3; sacculus with a small triangular bulge at basal 1/3 (Fig. 25c). Saccus digitate. Aedeagus (Fig. 26) slender, of even width throughout, narrower than saccus, apex oblique, slightly bent medially; with a zone of minute-spinulate cornuti 1/5 as long as aedeagus.

#### Female genitalia.

(Fig. 32) (2 preparations examined). S8 slightly oblique laterally, weakly sclerotized, with a pair of setose humps posteromedially; interspace between S8 humps with minute thorns; a pair of semicircular pleats lateroposteriorly (indicated with an asterisk in Fig. 32). Apophysis posterioris 4× longer than apophysis anterioris excluding basal Y-fork; longer branch of Y-fork 3× longer than shorter branch, 2.5× longer than apophysis anterioris. Ductus bursae 2× longer than corpus; antrum in posterior 1/6 of ductus bursae, cylindrical, with minute thorns on inner wall (Fig. 32a); ductus seminalis weakly sclerotized at connection with ductus bursae; bulla seminalis absent. Corpus bursae globular; signum absent.

#### Distribution.

Costa Rica (high elevations of Cartago Province).

#### Etymology.

The new species is named after Dr. Jerry A. Powell, director emeritus of the Essig Museum of Entomology, the University of California, Berkeley, in appreciation of his assistance with the first author's work.

**Figure 16. F4:**
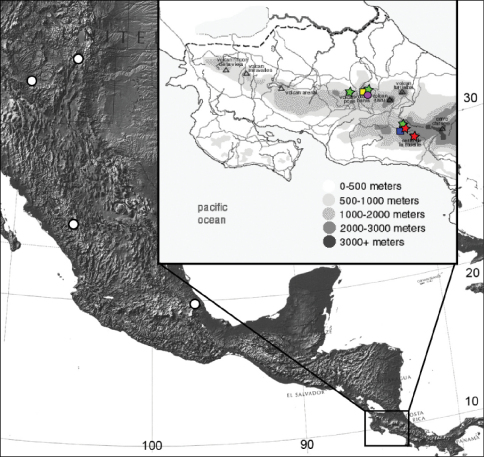
Distribution of *Eucalantica costaricae* sp. n. (red star), *Eucalantica ehecatlella* sp. n. (yellow box), *Eucalantica icarusella* sp. n. (green star), *Eucalantica powelli* sp. n. (blue box), *Eucalantica pumila* sp. n. (purple circle), and *Eucalantica vaquero* sp. n. (white circle). Maps from www.nationalatlas.gov and www.costa-rica-guide.com.

### Eucalantica pumila

Sohn sp. n.

urn:lsid:zoobank.org:act:CDA549BA-3817-4A91-A986-AEB63BCED19C

http://species-id.net/wiki/Eucalantica_pumila

Figs 13 29, 30 

#### Type material.

**Holotype** ♂ – COSTA RICA: Heredia, Volcan Barva, 6 km ENE from Vara Blanca, 10°11' N; 84°07'W, alt. 1950–2050 m, 20 February 2002, BN-INB0003219355, GSN [SJC 808] (INBIO).

#### Diagnosis.

This new species is easily distinguished from all other species of *Eucalantica* by its smaller size and in having a triangular, dark brown dorsal patch on the forewings. The male genitalia of *Eucalantica pumila* is similar to *Eucalantica costaricae*, but spines on the socii and the aedeagus is slender in the former.

#### Description

(Fig. 13).Forewing length 5.8 mm (n=1); costal streak on basal 1/10 of costal margin, black; dorsal patch subtriangular, dark brown, upper border extended to the lower side of the discal cell; terminal line with three dark brown dots between veins. Hindwing anterior margin 2.2× longer than maximum width, pale gray except dark gray apical area.

#### Male genitalia.

(Figs 29, 30). Uncus subpentagonal, convex posteriorly, with a papilliform projection apically; socii semielliptical, straight ventrally, 1.5× longer than saccus, long-hairy dorsally, with four slender terminal spines in a row, gradually smaller from basal to terminal spine (Fig. 29b). Tegumen subtrapezoidal; subscaphium (Fig. 29d) slightly bulged. Valva elongate, of even width throughout, narrowly rounded apically; costa slightly convex at basal 1/3; sacculus ending at basal 1/4 of ventral margin of valva; an arched setal area above basal area of sacculus (Fig. 29c). Saccus slender, 2× longer than uncus. Aedeagus (Fig. 30) slender, narrower in distal half, almost straight, obtuse terminally; a zone of minute-spinulate cornuti 1/2 as long as aedeagus.

#### Female.

unknown.

#### Distribution.

Costa Rica (only known from the type locality).

#### Etymology.

The specific epithet is derived from the Latin *pumilus*, meaning “little", and refers to its small size relative to other *Eucalantica*.

**Figures 17–24. F5:**
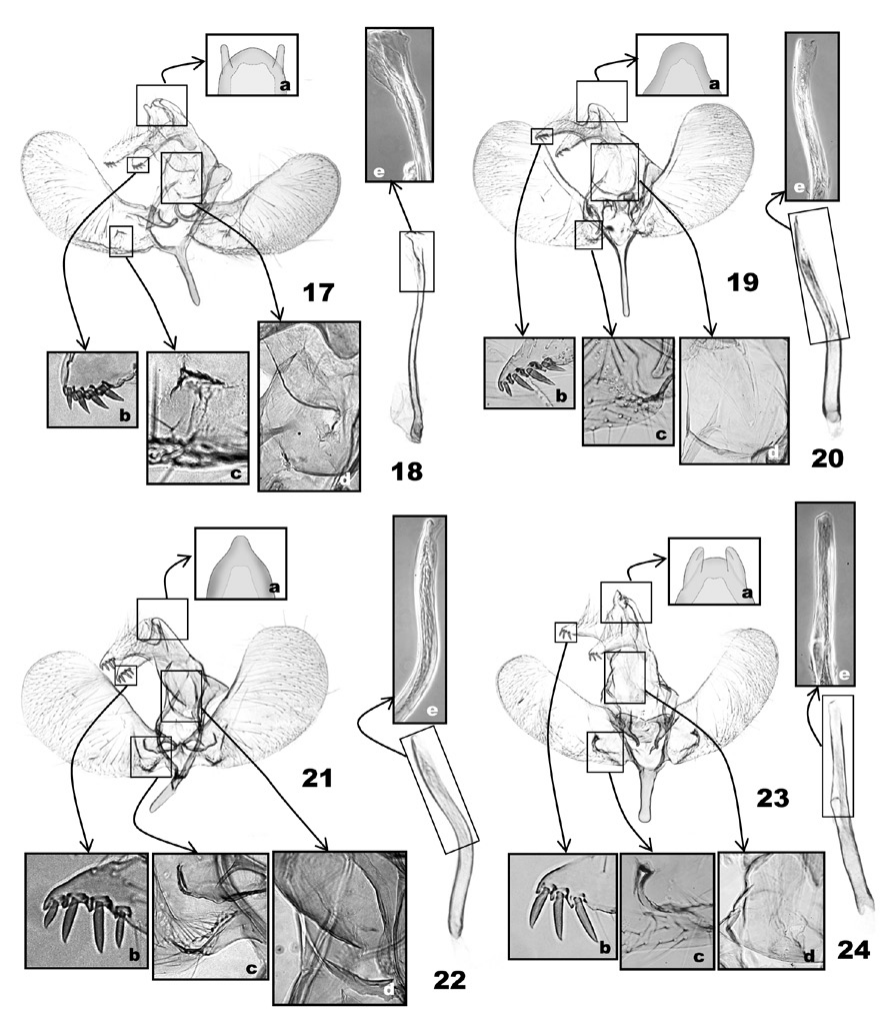
Male genitalia of *Eucalantica*. **17–18.** *Eucalantica polita* **19–20** *Eucalantica costaricae* sp. n. (holotype) **21–22** *Eucalantica ehecatlella* sp. n. (holotype) **23–24** *Eucalantica icarusella* sp. n. (holotype). **18, 20, 22, 24** aedeagus. Close-up boxes: a – apical region of uncus; b – terminal spines on socius; c – grooves or projections above sacculus; d – subscaphium; e – terminal part of aedeagus and cornuti (transmitted light phase contrast image).

### Eucalantica vaquero

Sohn sp. n.

urn:lsid:zoobank.org:act:C77690E1-7618-4E54-B802-19EFFCE72FFC

http://species-id.net/wiki/Eucalantica_vaquero

Figs 14 27–28 34 

#### Type material.

**Holotype** ♂ – USA: New Mexico, Pecos National Forest, 35°53'N, 105°38'W, alt. 3048 m, 24 August 1916, C Heinrich, GSN [USNM-96389] (USNM). Paratypes (2♂3♀) – USA: 1♀, New Mexico, same as holotype, abdomen missing (USNM). 1♂, Arizona, White Mts., Summit of Mt. Thomas, 33°54'22"N, 109°33'46"W, alt. 11500 ft, 20 August 1925, OC Poling (USNM). MEXICO: 1♀, Tepalcates, 48 km W from Durango, Dgo, 24°01'N, 104°40'W, alt. 2560 m, 4–8 August 1972, J Powell, D Veirs, & CD MacNeill, GSN [EMEC-JCS 011] (EMEC). 1♂, Veracruz, Cañón Las Minas, 13 km NE from Perote, 19°29'52"N, 97°52'09"W, alt. 2150 m, 19 August 1987, J Brown & J Powell (EMEC); 1♀, Veracruz, 7 km NW from Banderilla, 19°35'N, 95°56'W, alt. 1680 m, 13 July 1974, J Powell & J Chemsak (EMEC).

#### Diagnosis.

This new species is superficially indistinguishable from some variants of *Eucalantica polita* and in such cases, examination of the genitalia is necessary for a reliable identification. *Eucalantica vaquero* is also similar to *Eucalantica costaricae* in having a reduced dorsal patch on the forewings but differs from the latter by having the fewer black spots on the forewing, mainly around the CuP fold. The male genitalia of *Eucalantica vaquero* differ from ones of *Eucalantica polita* and *Eucalantica costaricae* in having a bulge on apex of the uncus and stouter saccus. In the female genitalia, *Eucalantica vaquero* is distinguished from the latter two in having keel-like signum in the corpus bursae.

#### Description

(Fig. 14). Forewing length 7.5–8.0mm (mean=7.65mm, n=4); costal streak on basal 1/3 narrow; dorsal patch reduced to a small, oblique, reddish brown band intermixed with black spots or absent; fringes white in basal 2/3, pale gray in distal 1/3. Hindwing anterior margin 2× longer than maximum width; fringes pale gray.

#### Male genitalia.

(Figs 27, 28) (4 preparations examined). Uncus (Fig. 27a) linguiform, bulged dorsoapically, lateral lobes upcurved, digitate; socii digitate, as long as saccus, long-hairy dorsally, with four terminal spines, all of them almost equal in size (Fig. 27b). Tegumen parallel laterally, 2× broader than uncus; tuba analis with minute thorns on inner wall; subscaphium (Fig. 27d) strongly bulged ventrad. Valva slightly broadened in distal half, narrowly round apically, saccular margin round in distal 1/3, almost straight in basal 2/3; costa slightly concave at middle; sacculus slightly bulged inward at basal 1/3; a semicircular setose area above saccular base; a longitudinal fold at base of valva, adjoining with a small dentiform process (Fig. 27c). Saccus digitate, robust. Aedeagus (Fig. 28) dillated at distal 1/3, almost straight; a zone of minute-spinulate cornuti 1/3 as long as aedeagus.

#### Female genitalia.

(Fig. 34) (2 preparations examined). S8 quadrate, sclerotized, with a pair of semicircular, setose humps. Minute thorns on S8 humps and an area connecting S8 humps and ostium bursae. Apophysis posterioris 4× longer than apophysis anterioris excluding basal Y-fork; both branches of Y-fork almost equal in length, 2× longer than apophysis anterioris. Ductus bursae 4/5 as long as corpus; antrum in posterior 1/4 of ductus bursa, conical, with minute thorns internally (Fig. 34a); bulla seminalis as long as ductus bursae; a sclerite at connection between bulla seminalis and ductus bursae (Fig. 34d). Corpus bursae ellipsoid; signum keel-like on middle of corpus, base narrow-elliptical, with a few denticles (Fig. 34c).

#### Distribution.

USA (New Mexico, Arizona) and Mexico.

#### Etymology.

The species name *vaquero* is a noun in apposition, meaning the Mexican cowboy, and refers to the distribution range of the new species roughly matching with the regions under ‘vaquero' traditions.

**Figures 25–30. F6:**
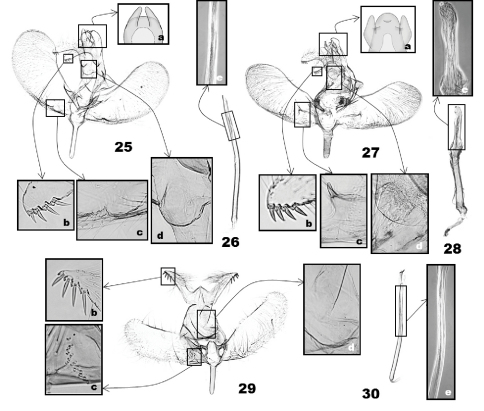
Male genitalia of *Eucalantica*. **25–26** *Eucalantica powelli* sp. n. (holotype) **27–28** *Eucalantica vaquero* sp. n. (holotype) **29–30** *Eucalantica pumila* sp. n. (holotype). **26, 28, 30** aedeagus. See figures **17–24** for close-up boxes.

**Figures 31–35.**
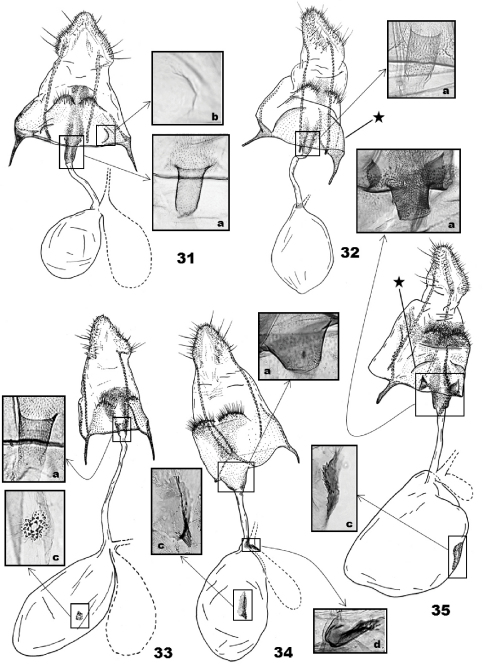
Female genitalia of *Eucalantica*. **31** *Eucalantica polita*. **32** *Eucalantica powelli* sp. n. (paratype) **33** *Eucalantica costaricae* sp. n. (paratype) **34** *Eucalantica vaquero* sp. n. (paratype) **35** *Eucalantica icarusella* sp. n. (paratype). Ductus seminalis and bulla seminalis contoured by dotted line. Asterisk = semicircular fold. Close-up boxes: a – antrum and thorny area around ostium; b – semicircular depression on eighth sternite; c – signum; d – sclerite at connection between ductus bursa and bulla seminalis.

## Supplementary Material

XML Treatment for Eucalantica

XML Treatment for Eucalantica polita

XML Treatment for Eucalantica costaricae

XML Treatment for Eucalantica ehecatlella

XML Treatment for Eucalantica icarusella

XML Treatment for Eucalantica powelli

XML Treatment for Eucalantica pumila

XML Treatment for Eucalantica vaquero
